# Identification of cuproptosis-related gene signature to predict prognosis in lung adenocarcinoma

**DOI:** 10.3389/fgene.2022.1016871

**Published:** 2022-10-14

**Authors:** Yanju Lv, Yajie Xiao, Xiaoli Cui, Haitao Luo, Long Xu

**Affiliations:** ^1^ Department of Internal Medicine, Second Affiliated College of Harbin Medical University, Harbin, China; ^2^ Department of Medicine, YuceBio Technology Co., Ltd., Shenzhen, China; ^3^ Department of Oncology, General Hospital of Northern Theater Command, Shenyang, China

**Keywords:** lung adenocarcinoma, cuproptosis, molecular subtype, RiskScore, prognosis

## Abstract

**Background:** Studies have reported that coppers are involved in the tumorigenesis and development of tumor. In herein, we aimed to construct a prognostic classification system for lung adenocarcinoma (LUAD) associated with cuproptosis.

**Methods:** Samples information of LUAD were acquired from The Cancer Genome Atlas (TCGA) and GSE31210 dataset. Cuproptosis-related genes were screened from previous research. ConsensusClusterPlus was applied to determine molecular subtypes, which evaluated by genome analysis, tumor immune microenvironment analysis, immunotherapy, functional enrichment analysis. Furthermore, univariate Cox analysis combined with Lasso analysis were employed to construct a cuproptosis-related risk model for LUAD.

**Results:** 14 genes related to cuproptosis phenotype were identified, and 2 clusters (C1 and C2) were determined. Among which, C1 had better survival outcome, less advanced stages, enhanced immune infiltration and enriched in TCA related pathways. A 7 cuproptosis-associated genes risk model was constructed, and the performance was verified in the GSE31210 dataset. A higher RiskScore was significantly correlated with worse overall survival, advanced stages. Cox survival analysis showed that RiskScore was an independent predictor. High-risk group patients had weakened immune infiltration, less likely to benefit from immunotherapy and was more sensitived to immunotherapy.

**Conclusion:** The cuproptosis-related gene signature could serve as potential prognostic predictors for LUAD patients and may provide clues for the intervention of cuproptosis induced harm and targeted anti-tumor application.

## Introduction

Lung adenocarcinoma (LUAD), the most common type of non-small cell lung cancer, is characterized by dense lymphocytic infiltration and early metastasis (Luo et al., 2020). Although treatment strategies for LUAD have improved greatly in recent years, the survival rate of patients with LUAD is still very low (Kleczko et al., 2019). Chemotherapy, surgical resection and radiotherapy are routine treatments for LUAD, However, due to the lack of specificity of these treatments, they can also cause damage to adjacent normal cells (Wang et al., 2021). Targeted therapy and immunotherapy are one of the main methods for the treatment of LUAD. Although both have achieved good clinical efficacy (Osmani et al., 2018; Xing et al., 2019), the clinical benefit population is still limited (Park and Jang, 2016; Testa et al., 2018). Therefore, it is of great significance to further search for new diagnostic markers and therapeutic targets for LUAD.

It is well known that copper, as a cofactor of essential enzymes, plays an important role in human life (Kim et al., 2008). The concentration of copper in normal cells is very low, which mainly prevents the harmful accumulation of free intracellular copper through the homeostasis mechanism across the concentration gradient, thus maintaining cellular copper homeostasis (Lutsenko, 2010; Ge et al., 2022). However, a recent study shows that copper death is dependent on mitochondrial respiration, which is different from the previously known programmed cell death (such as iron death and apoptosis). In this process, copper directly binds to the lipid components of the tricarboxylic acid cycle, resulting in the aggregation of lipoacylated proteins and the loss of iron-sulfur cluster proteins, leading to proteotoxic stress and cell death (Tsvetkov et al., 2022). The importance of copper homeostasis in immune infiltration has also been demonstrated in some recent correlation studies (Choi et al., 2013; Tan et al., 2021). Tan et al. (2021) found that copper chelation on macrophages can eliminate lysyl oxidase-like 4-mediated programmed death molecule ligand 1 presentation, thereby inhibiting cell immune escape. Choi et al. (2013) showed that chlorodoxyquine (a common copper chelator) can effectively reduce the infiltration of encephalitis-causing immune cells (CD4, CD8, etc.).

Based on this, this study is the first to investigate the prognosis of LUAD by combining LUAD microarray data and cuproptosis-related genes. Using the GSE31210 dataset of TCGA database and GEO database, combined with the 13 copper-death genes provided by Tsvetkov et al. (2022), we identified important cuproptosis related genes and molecular subtypes, and constructed a risk model. Finally, based on the subtypes and risk models obtained above, functional enrichment analysis, immune infiltration analysis, immunotherapy and chemotherapy drug prediction were performed, so as to provide some theoretical support for the mechanism research of cuproptosis in LUAD.

## Materials and methods

### Data source

In order to obtain the microarray data related to lung adenocarcinoma, TCGA and GEO databases were searched with “LUAD” as the search term. The TCGA-LUAD dataset contains 472 cancer patient samples and 59 healthy samples, and the GSE31210 dataset contains 226 LUAD samples. 472 tumor samples were classified into the training cohort randomly (*n* = 236), the testing cohort (*n* = 236). The two groups were similar in age, gender, Stage, follow-up time, and Event (Table 1).

**TABLE 1:** Sample information of TCGA training dataset and validation dataset.

Characteristics	Train (N = 236)	Test (N = 236)	Total (N = 472)	*p* value	FDR
Gender				1	1
FEMALE	127 (26.91%)	128 (27.12%)	255 (54.03%)		
MALE	109 (23.09%)	108 (22.88%)	217 (45.97%)		
T.stage				0.79	1
T1	76 (16.10%)	84 (17.80%)	160 (33.90%)		
T2	130 (27.54%)	123 (26.06%)	253 (53.60%)		
T3	22 (4.66%)	21 (4.45%)	43 (9.11%)		
T4	8 (1.69%)	7 (1.48%)	15 (3.18%)		
Ukown	0 (0.0e+0%)	1 (0.21%)	1 (0.21%)		
N.stage				0.2	1
N0	160 (33.90%)	153 (32.42%)	313 (66.31%)		
N1	40 (8.47%)	46 (9.75%)	86 (18.22%)		
N2	34 (7.20%)	28 (5.93%)	62 (13.14%)		
N3	0 (0.0e+0%)	2 (0.42%)	2 (0.42%)		
Ukown	2 (0.42%)	7 (1.48%)	9 (1.91%)		
M.stage				0.88	1
M0	159 (33.69%)	161 (34.11%)	320 (67.80%)		
M1	9 (1.91%)	7 (1.48%)	16 (3.39%)		
Ukown	68 (14.41%)	68 (14.41%)	136 (28.81%)		
Stage				0.83	1
I	135 (28.60%)	129 (27.33%)	264 (55.93%)		
II	53 (11.23%)	61 (12.92%)	114 (24.15%)		
III	36 (7.63%)	34 (7.20%)	70 (14.83%)		
IV	9 (1.91%)	7 (1.48%)	16 (3.39%)		
Ukown	3 (0.64%)	5 (1.06%)	8 (1.69%)		
Event				0.57	1
Alive	141 (29.87%)	148 (31.36%)	289 (61.23%)		
Dead	95 (20.13%)	88 (18.64%)	183 (38.77%)		
Age				0.13	0.92
<=65	69 (14.62%)	77 (16.31%)	146 (30.93%)		
>65	159 (33.69%)	157 (33.26%)	316 (66.95%)		
Ukown	8 (1.69%)	2 (0.42%)	10 (2.12%)		

The 13 cuproptosis related genes, SLC31A1, PDHB, PDHA1, LIPT1, FDX1, DLD, DLST, DBT, LIAS, DLAT, GCSH, ATP7A, and ATP7B, were derived from a recent report by team Tsvetkov et al. (2022).

### Differentially expressed genes analysis

Based on 13 cuprotosis related genes, scores of cuprotosis related genes in each sample were calculated by single sample gene set enrichment analysis (ssGSEA), and DEGs were screened between cancer tissues and para-carcinoma tissue with FDR<0.05 and |log2FC|>2.

Then, the correlation analysis between DEGs and scores were analyzed by pearson methods with selection criteria |R|>0.2 and *p*.value < 0.05 to obtained genes associated with cuproptosis phenotype.

### Univariate COX survival analysis

Next, Univariate COX survival analysis using coxph function of R package was used to analysis genes associated with cuproptosis phenotype with *p* < 0.05 to determine cuproptosis-related genes for LUAD prognosis, for subsequent analysis

### Cluster analysis

Base on cuproptosis-related genes, Then, molecular subtypes were performed separately for TCGA-LUAD dataset samples *via* the Consensus Cluster Plus 1.52.0 (Wilkerson and Hayes, 2010). “pam” arithmetic and “pearson” distance were utilized to complete 500 bootstraps with every bootstrap having specimens (≥80%) of TCGA-LUAD dataset. Cluster number k was between 2 and 10, and the optimum k was identified as per cumulative distribution function (CDF) and AUC. Survival curves (KM curves) between molecular subtypes were then analyzed for difference. In addition, differences in the distribution of clinical characteristics between molecular subtypes were compared and a chi-square test was completed, and *p* < 0.05 had significance on statistics.

### Single-sample GSEA

The ssGSEA was used to evaluate the various pathways scores (Charoentong et al., 2017) using GSVA of R package. NES>0 indicates pathway activation, and NES<0 indicates pathway inhibition.

### Estimation of STromal and immune cells in MAlignant tumours using expression data

R software ESTIMATE arithmetic (Yang et al., 2021) was utilized to compute overall stroma level (Stromal Score), the immunocyte infiltration (Immune Score) and the combination (ESTIMATE Score) of sufferers in the TCGA-LUAD cohort using Wilcox.test analysis to determine difference.

### Cell-type identification by estimating relative subsets of RNA transcripts

CIBERSORT analyses were utilized to compare diversities in different immunocytes in molecular subtypes. Wilcox.test analyses were completed to identify the difference of 22 kinds of infiltrating immunocyte score between molecular subtypes. The “ggplot2” package (Ito and Murphy, 2013) was used to realize the visualization of the distributional status of the diversities in 22 kinds of infiltration immunocytes.

### Immunotherapy

The expression levels of 47 immune checkpoint genes, which from HigsAtlas (Liu et al., 2017), were determined.

### Construction and evaluation a prognostic risk model for lung adenocarcinoma

Lasso-cox regression was performed using the Glnmet package in R language to select the best prognostic genes (Tibshirani, 1997). Glmnet is a software package for fitting generalized linear and similarity models by penalized maximum likelihood. The regularization path is the calculation of the lasso or elastic net penalty on the value (on a logarithmic scale) of the regularization parameter lambda (Goeman, 2010). The optimal value of the penalty coefficient λ and the genes to be included in the model were selected by running the 10-fold cross-validation probability 1000 times. Subsequently, Cox multivariate regression analysis coefficients of prognostic genes were extracted, and the gene expression levels were used to calculate the risk score by the following formula as the survival risk score of each patient:

$$RiskScore=\overset{n}{\underset{k=0}{\sum }}\beta i\times Expi$$

Where, βi represents the Cox hazard ratio coefficient of mRNA, and Expri represents the gene expression level. TCGA-LUAD samples were divided into high risk (RiskScore>0) and low risk groups (RiskScore<0) according to the risk score, which was for zscore. At the same time, GSE31210 were used to evaluate the effectiveness and robustness of the prognostic risk model. Kaplan-Meier (KM) curves combined with the Logrank test were used to analyze survival differences among different risk groups. The timeROC package was used to determine the area under the receiver operating characteristic curve (AUC) to predict 1-year, 2-year, 3-year, 4-year and 5-year survival rates, respectively.

### Independent prognostic power of RiskScore

Univariate and multivariate COX regression were used to examine the independent prognostic power of RiskScore.

### Tumor immune dysfunction and exclusion

TIDE (Jiang et al., 2018; Fu et al., 2020) algorithm (http://tide.dfci.harvard.edu) was used to evaluate three cell types that limit T-cell invasion into tumors, including IFNG, myeloid suppressor cells (MDSC), and M2 subtypes of tumor-associated macrophages (TAM.M2), as well as dysfunction of tumor infiltration cytotoxic T lymphocytes (CTL) and exclusion of CTL by immunosuppressive factors.

### Chemotherapy drugs sensitivity analysis

pRRophetic (Geeleher et al., 2014) was used to predict the sensitivity of Cisplatin, Salubrinal, Vinorelbine, QS11, AKT inhibitor ⅤⅢ and Embelin to IC50.

Sangerbox provided assistance with this article (Shen et al., 2022).

## Results

### Identification of genes closely related to cuproptosis related gene pathway score

**FIGURE 1:**
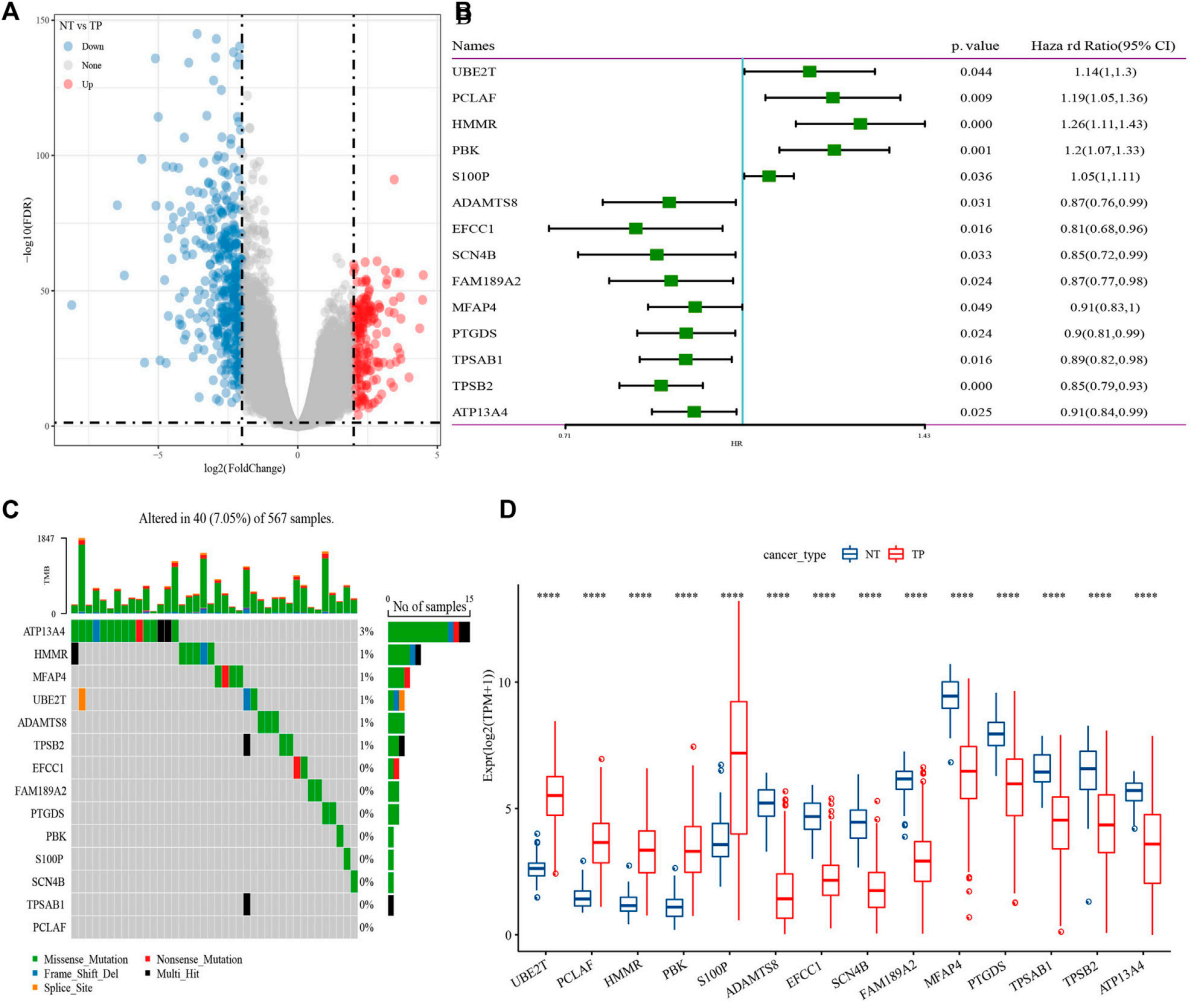
Identification of genes closely related to cuproptosis related gene pathway score. **(A)** Differentially expressed gene between cancer tissue and para-carcinoma tissue. **(B)** 14 genes closely related to cuproptosis related gene pathway score. **(C)** mutation analysis of genes in TCGA-LUAD dataset. **(D)** The expression levels of 14 genes in cancer tissue and para-carcinoma tissue. ***p < 0.0001.

602 DEGs were screened between cancer tissue and para-carcinoma tissue in TCGA-LUAD dataset (Figure 1A), from which, 138 genes were closely with cuproptosis related gene pathway score. Next, Univariate Cox regression analysis identified 14 genes associated with prognosis in lung adenocarcinoma (Figure 1B). 40 of 567 samples (7.05%) in TCGA-LUAD had genes mutation (Figure 1C). The expression levels of 14 genes had significance between cancer tissue and para-carcinoma tissue (Figure 1D). Those data showed that cuproptosis was associated with LUDA.

### Identification of molecular subtypes

**FIGURE 2:**
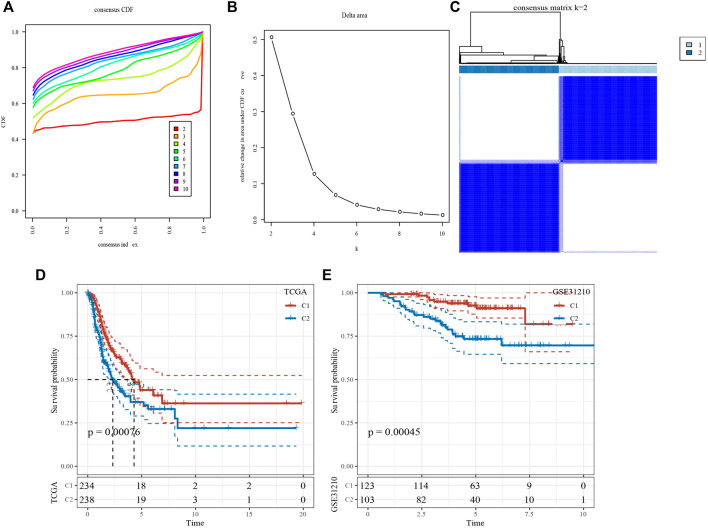
Identification of molecular subtypes. **(A)** Cumulative distribution function. **(B)** Delta area. **(C)** Heatmap of sample clustering when k = 2. **(D)** KM survival analysis of C1 and C2 in TCGA-LUAD dataset. **(E)** KM survival analysis of C1 and C2 in GSE31210 dataset.

**FIGURE 3:**
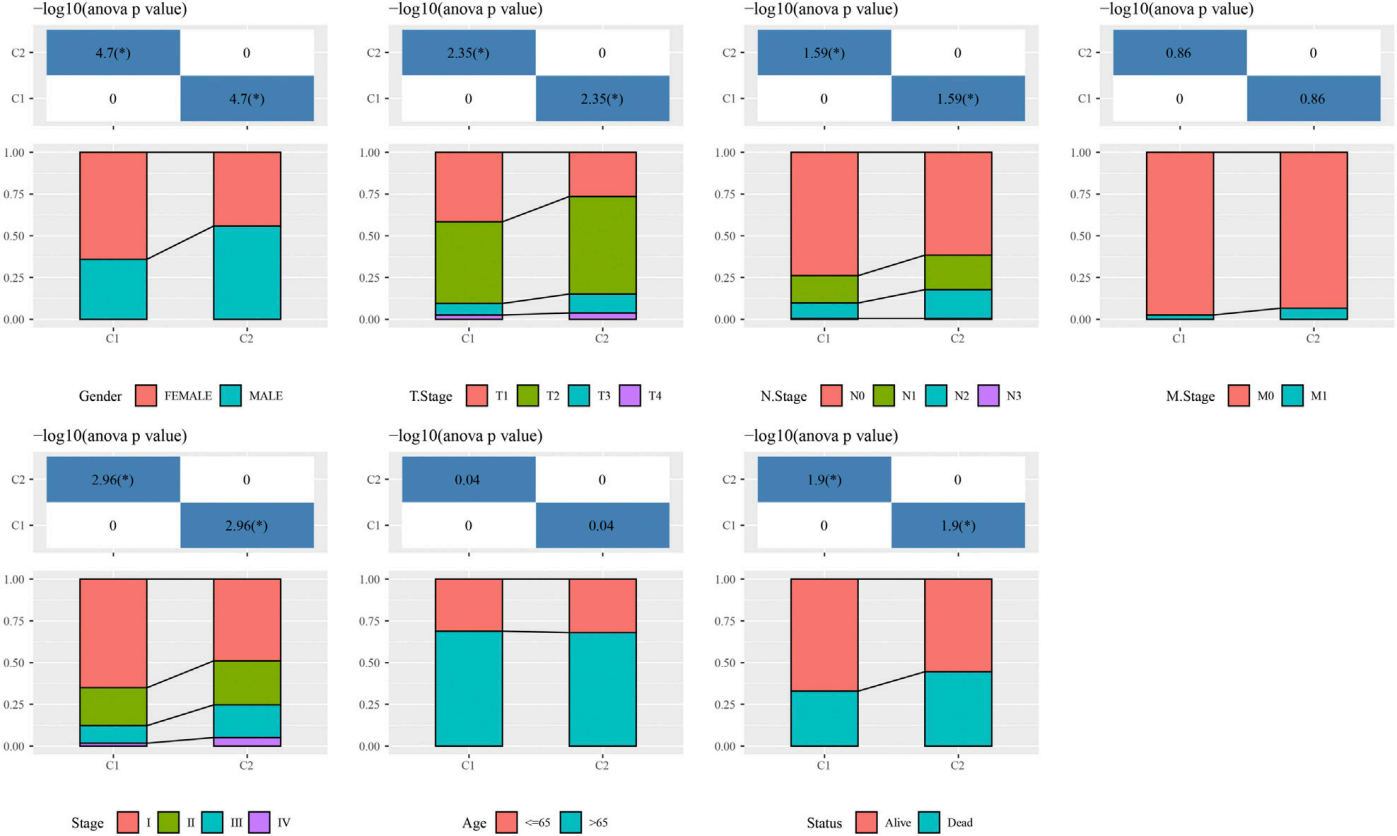
The distribution of clinical features, included Gender, T Stage, N Stage, M Stage, Stage Ages, and Status in C1 and C2. *p < 0.05.

Based on 14 genes, samples in TCGA-LUAD dataset were clustered with CDF and delta area (Figures 2A,B). When k = 2, 2 clusters (C1 and C2) were found (Figure 2C). KM survival analysis indicated that patients in C1 had better survival outcome in TCGA-LUAD dataset (*p* = 0.00076, Figure 2D) and GSE32210 dataset (*p* = 0.00045, Figure 2E). Distribution of clinical features in clusters showed that samples in C2 had more Male, T3/4 stage, N1/2 stage, StageⅢ/Ⅳ and Dead patients (Figure 3). Those analysis indicated that the 2 clusters had clinical significance.

### High gene mutation was observed in subtypes

**FIGURE 4:**
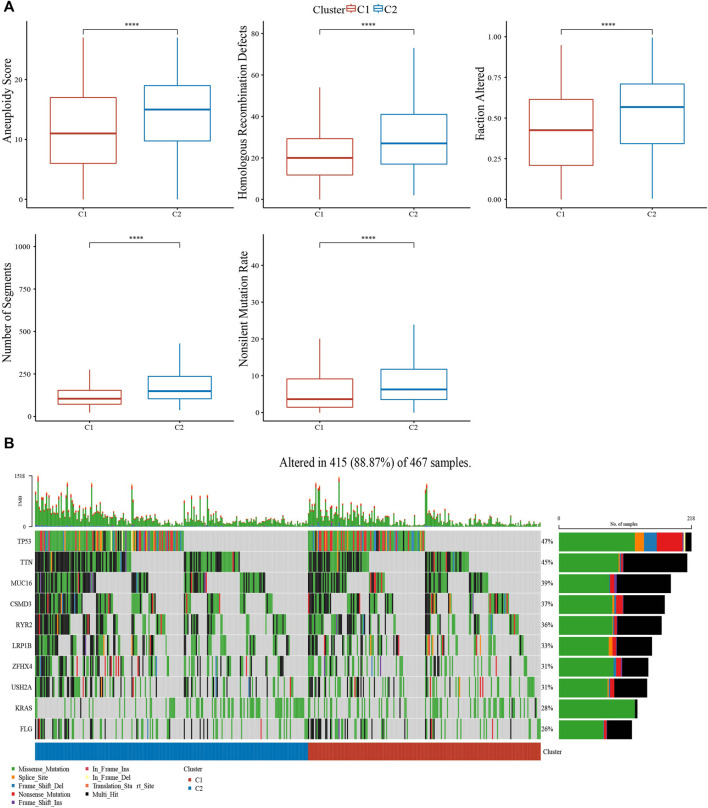
Genome analysis. **(A)** the analysis of Aneuploidy Score, Homologous Recombination Defects, Fraction Altered, number of segments, and non-silent mutation rate in C1 and C2. **(B)** Top 10 mutation genes in C1 and C2. ***p < 0.0001.

Genome analysis between clusters showed that C1 patients presented less Aneuploidy Score, Homologous Recombination Defects, Fraction Altered, Number of Segments, Nonsilent Mutation Rate (Figure 4A). In addition, top10 genes, especially TP53, TTN, MUC16, had obviously mutation differences between C1 and C2 (Figure 4B).

**FIGURE 5:**
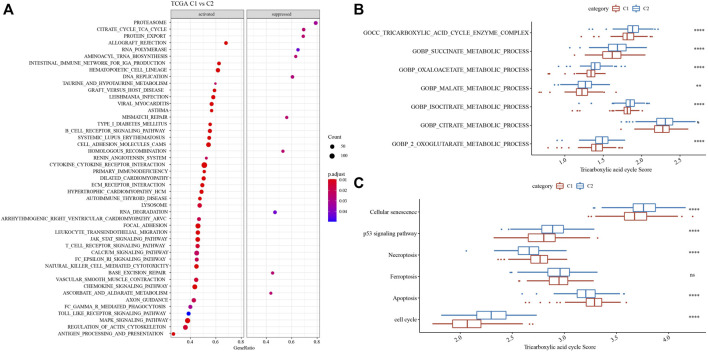
Functional enrichment analysis. **(A)** GSEA analysis demonstrated that pathways, such as, cell cycle were activated in C2. **(B)** 7 TCA pathways were activated in C2. **(C)** 6 pathways associated with tumorigenesis had differences in C1 and C2. *p < 0.05, **p < 0.01, ***p < 0.0001, ns: no significance.

Moreover, GSEA analysis showed that such as CITRATE_CYCLE_TCA_CYCLE and AMINOACYL_TRNA_BIOSYNTHESIS were activated in C2, while, TAURINE_AND_HYPOTAURINE_METABOLISM were activated in C1 (Figure 5A). Tricarboxylic acid cycle related pathways and genes were acquired to calculated TCA pathways scores using ssGSEA, and the results showed that 7 pathways were higher enriched in C2 (Figure 5B). Cell growth and death pathways, and genes were obtained from Kyoto Encyclopedia of Genes and Genomes (KEGG) (https://www.kegg.jp/kegg/pathway.html), ssGSEA analysis indicated that cellular senescence, p53 signaling pathway and cell cycle were higher in C2, while Necroptosis and Apoptosis were activated in C1 (Figure 5C).

### C1 had higher immune infiltration

**FIGURE 6:**
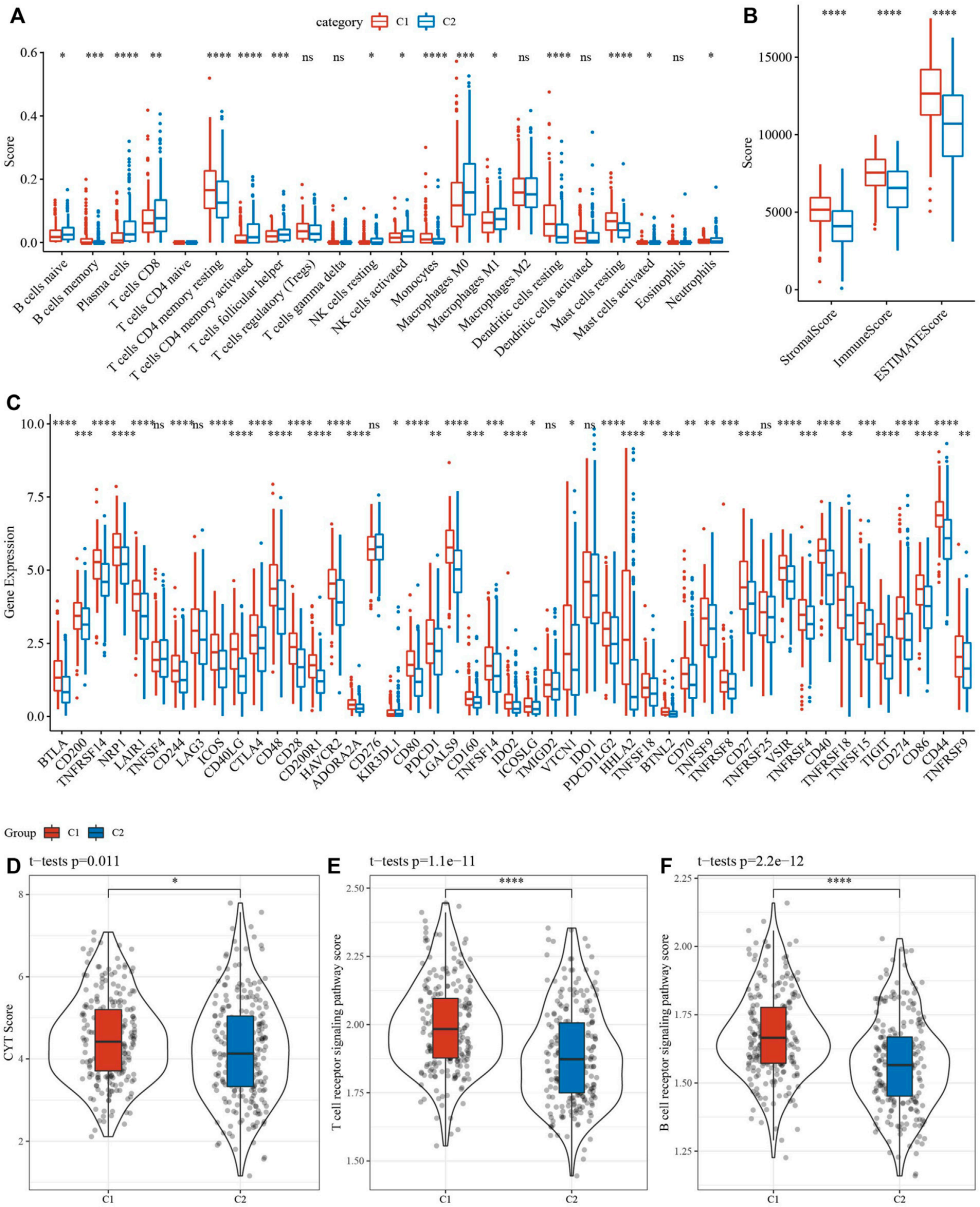
Analysis of immune infiltration. **(A)** analysis of 22 immune cells using CIBERSORT. **(B)** Analysis of immune infiltration using ESTIMATE. **(C)** The expression levels of 42 immune check genes between C1 and C2. **(D–F)** The differences of CTY score, T cell receptor signaling pathway score, B cell receptor signaling pathway score between C1 and C2. *p < 0.05, **p < 0.01, ***p < 0.001, ***p < 0.0001, ns: no significance.

16 of 22 immune cells had significantly difference using CIBERSORT analysis between 2 clusters (Figure 6A). Then, ESTIMATE analysis showed that C1 had higher score of StromalScore, ImmuneScore and ESTIMATEScore (Figure 6B). Our team afterwards evaluated the 47 immune check genes expressions, and 41 immune checkpoint genes had obviously high expressions in C1 that those in C2 (Figure 6C). Next, the scores of CYT, T cell receptor signaling pathway and B cell receptor signaling pathway, were calculated using ssGSEA, and they all were higher in C1 that those in C2 (Figures 6D–F).

### Identification of hub genes and RiskScore

**FIGURE 7:**
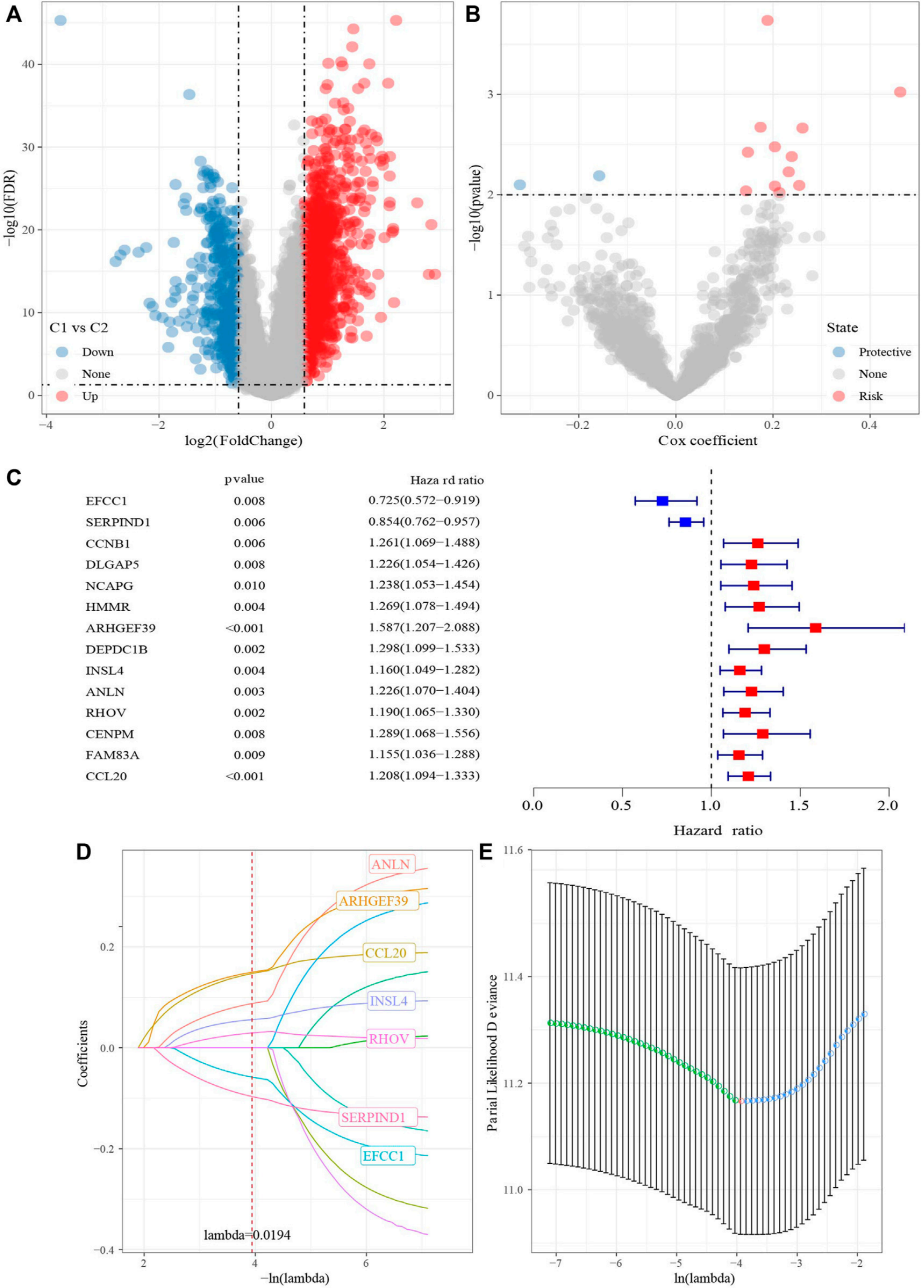
Identification hub cuproptosis related genes. **(A)** Volcano of differentially expressed genes identified from C1 and C2. **(B)** Volcano of differentially expressed genes identified using univariate Cox analysis. **(C)** Forest map of differentially expressed genes identified using univariate Cox analysis. **(D)** Lambda trajectory of differentially expressed genes. **(E)** Confidence interval under lambda.

1687 DEGs, including 1462 upregulated genes and 422 downregulated genes, were identified in C1 vs. C2 (Figure 7A). TCGA-LUAD dataset was divided into TCGA- training dataset and TCGA-test dataset. In TCGA- training dataset, univariate Cox survival analysis determined 14 genes associated with prognosis, included 12 risk genes and 2 protective genes (Figures 7B,C). LASSO Cox regression module was conducted to build a prognostic signature based on the expression matrix of the 14 genes. Consequently, we identified a 7-genes signature module according to the optimal λ value (Figures 7D,E). RiskScore of LUAD patients base on 7 genes was calculated using the following formula: RiskScore = 0.168*ARHGEF39-0.079*EFCC1-0.124*SERPIND1+0.065*INSL4+0.11* ANLN+0.04*RHOV+0.17*CCL20.

### Prognostic model has well predictive performance

**FIGURE 8:**
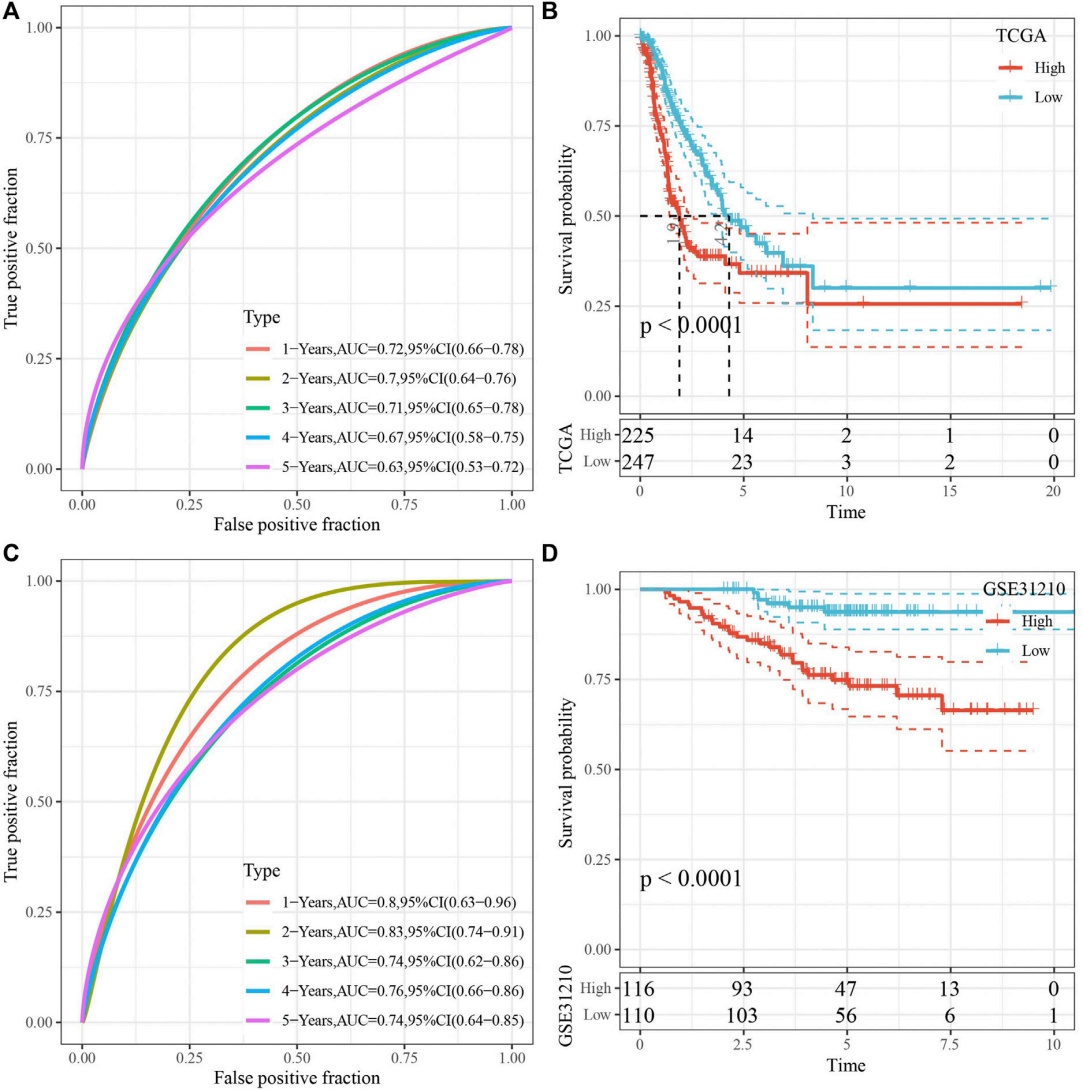
Validation of RiskScore. **(A)** ROC analysis of RiskScore in TCGA-test dataset. **(B)** KM survival analysis of RiskScore in TCGA-test dataset. **(C)** ROC analysis of RiskScore in GSE31210 dataset. **(D)** KM survival analysis of RiskScore in GSE31210 dataset.

**FIGURE 9:**
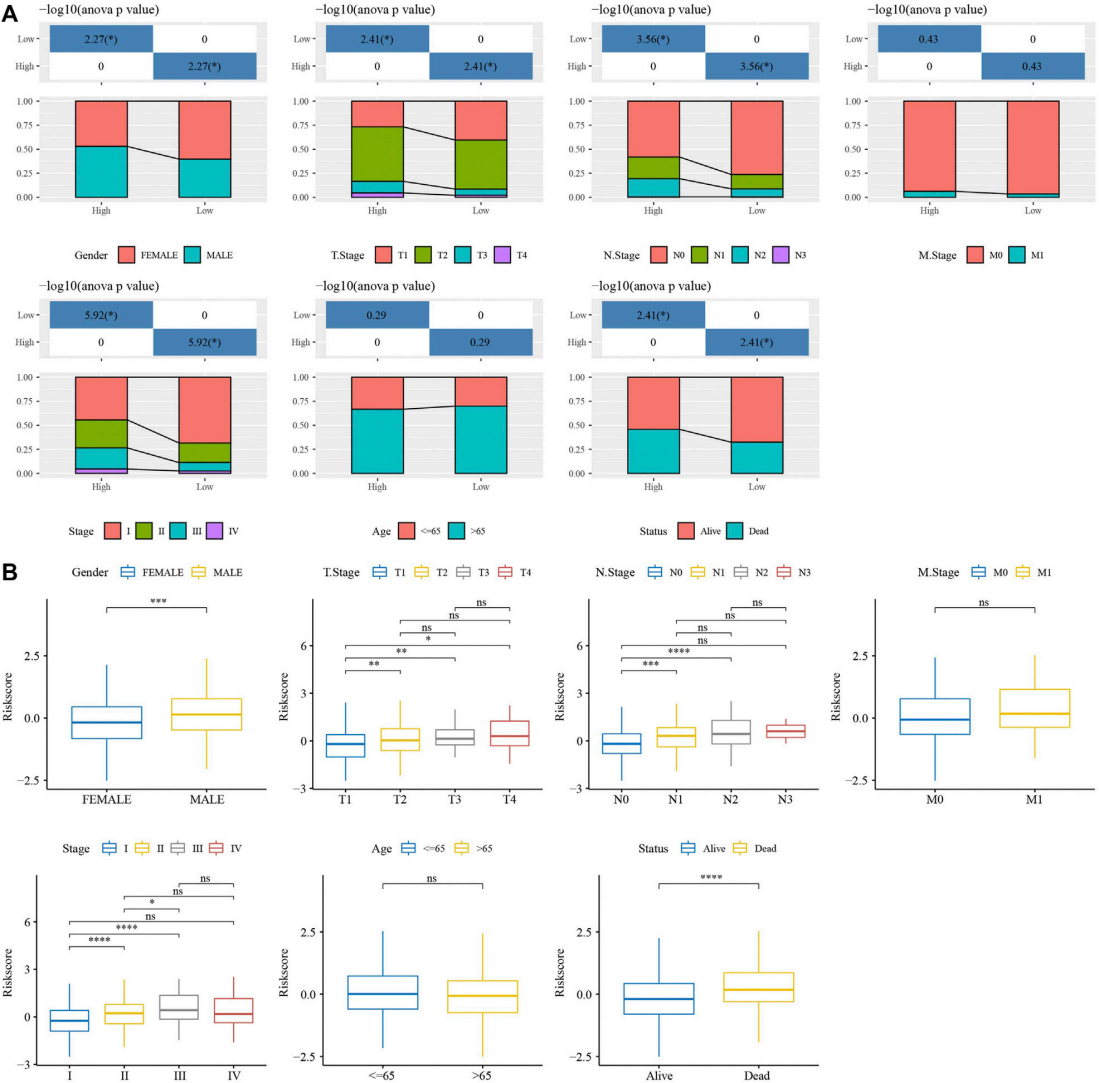
Analysis of clinical features in RiskScore. **(A)** The distribution of clinical features groups, included Gender, T Stage, N Stage, M Stage, Stage, Age and Status, in high group and low group. **(B)** The RiskScore differences analysis in clinical features groups, included Gender, T Stage, N Stage, M Stage, Stage, Age and Status. *p < 0.05, **p < 0.01, ***p < 0.001, ***p < 0.0001, ns: no significance.

RiskScore was for zscore, and the samples into high-risk (RiskScore> 0) and low-risk (RiskScore <0) groups in TCGA-test and GSE31210 dataset. ROC and survival analyses were performed in TCGA-test dataset (Figures 8A,B) and GSE31210 dataset (Figures 8C,D). The results revealed that the accuracy of the model was better in predicting the 1‐, 2-, 3‐, 4-, and 5‐year survival rates in above datasets, as all values of the area under the curve (AUC) were greater than 0.6. Results of Kaplan-Meier survival analysis showed overall survival was higher in low-risk group than high-risk group. High group had more samples with higher clinical grade (Figure 9A), the RiskScore was higher in MALE, a higher T stage, N2 stage and clinical stage, and dead samples (Figure 9B).

### RiskScore was an independent prognostic factor

**FIGURE 10:**
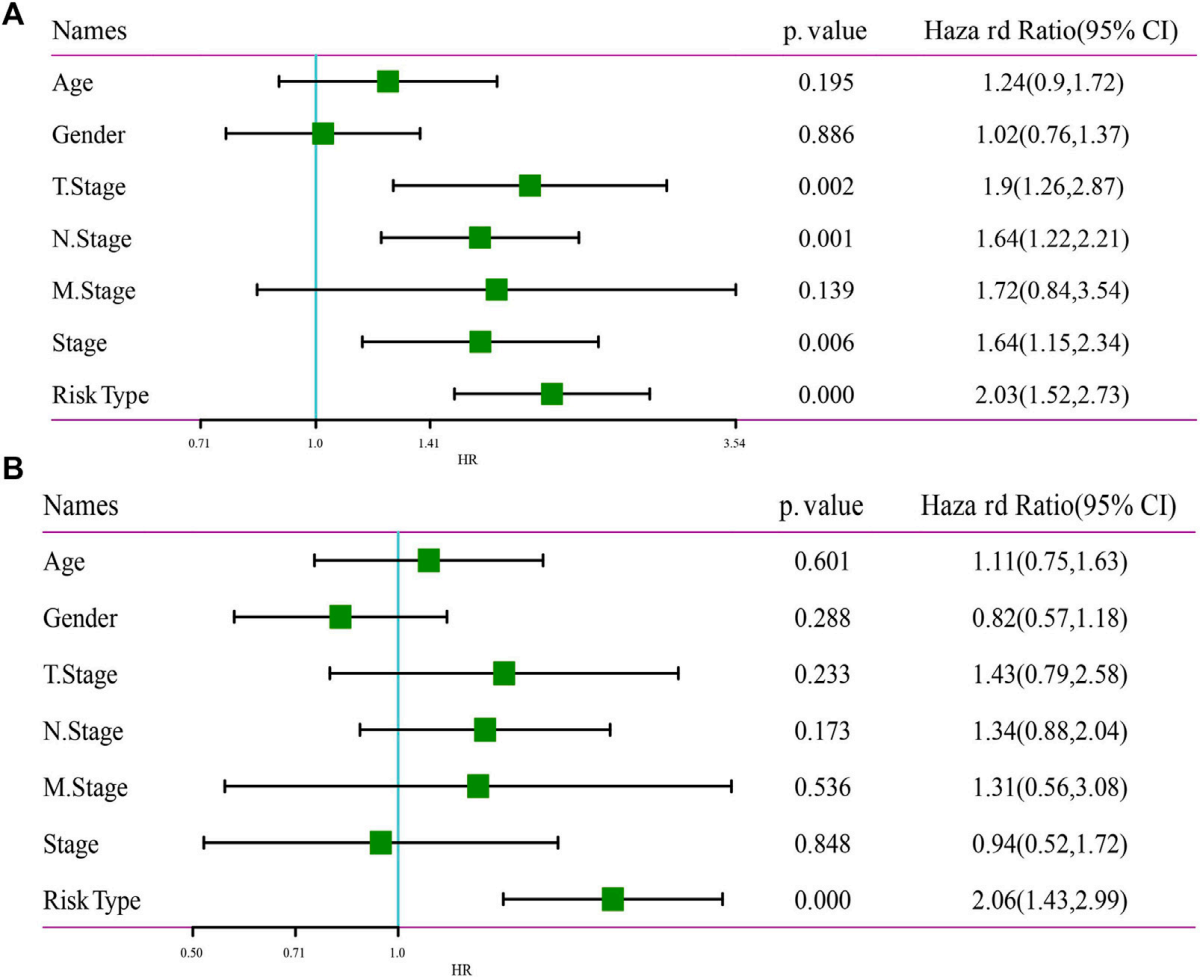
Independence of RiskScore. **(A)** Univariate Cox regression analysis. **(B)** Multivariate Cox regression analysis.

To identify the independence of 7-gene signature model in clinical application, in TCGA-LUAD dataset, univariate and multivariate COX regression were used to analyze the HR, 95%CI of HR and Pvalue of Age, Gender, T Stage, N Stage, M Stage, Stage and RiskType. Univariate COX regression analysis showed that T Stage, N Stage, Stage and RiskType were significantly associated with survival (Figure 10A), while multivariate COX regression analysis showed that only RiskType (HR = 2.06, 95%CI = 1.43–2.99, *p* < 0.001) was still significantly associated with survival (Figure 10B). Those data imply that RiskType was an independent prognostic factor.

### Low group had higher immune infiltration and sensitived to immunotherapy

**FIGURE 11:**
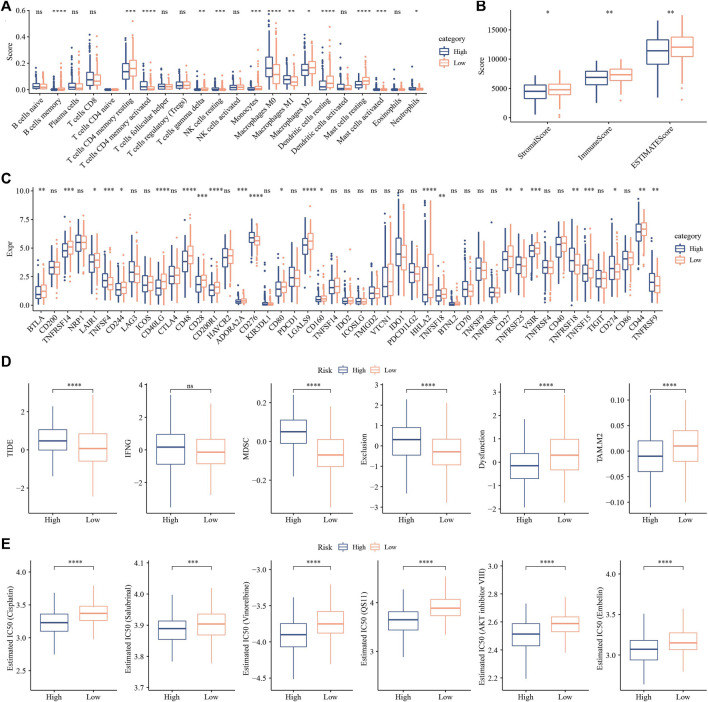
Analysis of immune infiltration. **(A)** analysis of 22 immune cells using CIBERSORT. **(B)** Analysis of immune infiltration using ESTIMATE. **(C)** The expression levels of 42 immune check genes between low group and high group. **(D)** The differences of TIDE, IFNG, MDSC, Exclusion, Dysfunction and TAM.M2 between low group and high group. **(E)** IC50 of traditional drugs in low group and high group. *p < 0.05, **p < 0.01, ***p < 0.001, ***p < 0.0001, ns: no significance.

CIBERSORT analysis indicated that 14 of 22 immune cells, most were significantly higher in low group that those in high group (Figure 11A). While, ESTIMATE analysis showed that low group had higher StromalScore, ImmuneScore and ESTIMATEScore (Figure 11B). And 24 immune checkpoint genes had obviously difference expressions between high group and low group (Figure 11C). TIDE, MDSC and Exclusion were lower in low group that in high group, while Dysfunction and TAM.M2 were higher in low group (Figure 11D), suggesting that low group was more likely to benefit from immunotherapy. IC50 of Cisplatin, Salubrinal, Vinorelbine, QS11, AKT inhibitor ⅤⅢ and Embelin were higher in low group, which suggested the developed model could be used to predict chemotherapeutic drug sensitivity (Figure 11E).

## Discussion

Cuproptosis is a newly discovered form of cell death, which is characterized by the accumulation of intracellular free copper and the lipidation of proteins leading to cytotoxic stress, thereby inducing cell death (Tsvetkov et al., 2022). However, the mechanism of copper death in LUAD has not been studied. Based on this, the relevant microarray was downloaded from TCGA and GEO databases, and the correlation and difference of immune infiltration were analyzed. Then, the results were integrated with cuproptosis related genes, and the risk model was constructed. Finally, seven copper death genes related to lung adenocarcinoma were screened out, including ARHGEF39, EFCC1, SERPIND1, INSL4, ANLN, RHOV and CCL20.

The overexpression of ARHGEF39 has also been identified in various human malignancies, including non-small cell lung cancer (Zhou et al., 2018), gastric cancer (Wang et al., 2018), and hepatocellular carcinoma (Wang et al., 2012). Decreased expression of EFCC1 was significantly associated with progression of LUAD (Xia et al., 2019; Yu and Zhang, 2020). SERPIND1 acts as a potential oncogene in the development of tumor, including in lung cancer (Bossé et al., 2012; Zhu et al., 2016). INSL4 as prognostic marker for proliferation and invasiveness in Non-Small-Cell Lung Cancer (Scopetti et al., 2021). ANLN participates in cell developmental processes *via* regulating nuclear division pathway in LUAD (Long et al., 2018). Overexpression of RHOV in LUAD promotes the progression (Chen et al., 2021). Production of CCL20 from lung cancer cells induces the cell migration and proliferation (Wang et al., 2016). To sum up, although the copper death related gene in LUAD mechanism study is less, but according to previous research and the research results can be speculated that cuproptosis related genes may play an important role in LUAD progress, steady state and how to adjust the copper to prevention and treatment of LUAD, is the need for further research.

The analysis results of this study have certain reference value for the subsequent basic research of cuproptosis on LUAD, and could reduce unnecessary waste in experiments to a certain extent. However, this study still has some limitations. First, although the chip data used has met the sample size required by the research, the results may still be biased due to the small sample size. Second, although cuproptosis related genes associated with LUAD have been screened out, their specific mechanism of action has not been elucidated, which needs to be further explored in subsequent studies.

## Data Availability

The datasets presented in this study can be found in online repositories. The names of the repository/repositories and accession number(s) can be found in the article/Supplementary Material.
