# Supplementary material for: Identification of cuproptosis-related gene signature to predict prognosis in lung adenocarcinoma
Source: Front Genet. 2022 Oct 14;13:1016871. doi: 10.3389/fgene.2022.1016871 (PMC9614324; doi:10.3389/fgene.2022.1016871)
Supplement: Supplementary file 1 [file Table1.DOCX]

For the data analyzed in this study please see:

https://www.jianguoyun.com/p/DfBnZjUQzaXrChi8vdAEIAA
