# Supplementary material for: Identification of cuproptosis-related gene signature to predict prognosis in lung adenocarcinoma
Source: Front Genet. 2022 Oct 14;13:1016871. doi: 10.3389/fgene.2022.1016871 (PMC9614324; doi:10.3389/fgene.2022.1016871)
Supplement: Supplementary file 2 [file Table2.DOCX]

**Table 1. Sample information of TCGA training dataset and validation dataset**

| **Characteristics** | **Train(N=236)** | **Test(N=236)** | **Total(N=472)** | **pvalue** | **FDR** |
| --- | --- | --- | --- | --- | --- |
| **Gender** |  |  |  | 1 | 1 |
| FEMALE | 127(26.91%) | 128(27.12%) | 255(54.03%) |  |  |
| MALE | 109(23.09%) | 108(22.88%) | 217(45.97%) |  |  |
| **T.stage** |  |  |  | 0.79 | 1 |
| T1 | 76(16.10%) | 84(17.80%) | 160(33.90%) |  |  |
| T2 | 130(27.54%) | 123(26.06%) | 253(53.60%) |  |  |
| T3 | 22(4.66%) | 21(4.45%) | 43(9.11%) |  |  |
| T4 | 8(1.69%) | 7(1.48%) | 15(3.18%) |  |  |
| Ukown | 0(0.0e+0%) | 1(0.21%) | 1(0.21%) |  |  |
| **N.stage** |  |  |  | 0.2 | 1 |
| N0 | 160(33.90%) | 153(32.42%) | 313(66.31%) |  |  |
| N1 | 40(8.47%) | 46(9.75%) | 86(18.22%) |  |  |
| N2 | 34(7.20%) | 28(5.93%) | 62(13.14%) |  |  |
| N3 | 0(0.0e+0%) | 2(0.42%) | 2(0.42%) |  |  |
| Ukown | 2(0.42%) | 7(1.48%) | 9(1.91%) |  |  |
| **M.stage** |  |  |  | 0.88 | 1 |
| M0 | 159(33.69%) | 161(34.11%) | 320(67.80%) |  |  |
| M1 | 9(1.91%) | 7(1.48%) | 16(3.39%) |  |  |
| Ukown | 68(14.41%) | 68(14.41%) | 136(28.81%) |  |  |
| **Stage** |  |  |  | 0.83 | 1 |
| I | 135(28.60%) | 129(27.33%) | 264(55.93%) |  |  |
| II | 53(11.23%) | 61(12.92%) | 114(24.15%) |  |  |
| III | 36(7.63%) | 34(7.20%) | 70(14.83%) |  |  |
| IV | 9(1.91%) | 7(1.48%) | 16(3.39%) |  |  |
| Ukown | 3(0.64%) | 5(1.06%) | 8(1.69%) |  |  |
| **Event** |  |  |  | 0.57 | 1 |
| Alive | 141(29.87%) | 148(31.36%) | 289(61.23%) |  |  |
| Dead | 95(20.13%) | 88(18.64%) | 183(38.77%) |  |  |
| **Age** |  |  |  | 0.13 | 0.92 |
| <=65 | 69(14.62%) | 77(16.31%) | 146(30.93%) |  |  |
| >65 | 159(33.69%) | 157(33.26%) | 316(66.95%) |  |  |
| Ukown | 8(1.69%) | 2(0.42%) | 10(2.12%) |  |  |
|  |  |  |  |  |  |
